# Parametric Characterization of Machined Textured Surfaces

**DOI:** 10.3390/ma16010163

**Published:** 2022-12-24

**Authors:** Pawel Pawlus, Rafal Reizer, Michal Wieczorowski

**Affiliations:** 1 Faculty of Mechanical Engineering and Aeronautics, Rzeszow University of Technology, Powstancow Warszawy 8 Street, 35-959 Rzeszow, Poland; 2 Institute of Materials Engineering, College of Natural Sciences, University of Rzeszow, Pigonia Street 1, 35-310 Rzeszow, Poland; 3 Division of Metrology and Measurement Systems, Faculty of Mechanical Engineering, Institute of Mechanical Technology, Poznan University of Technology, Piotrowo Street 3, 60-965 Poznan, Poland

**Keywords:** surface texture, textured surfaces, two-process surfaces, parameters, correlation, regression

## Abstract

Surface topography in general is not easy to characterize due to a great number of different features that appear on it. It is still more challenging for machined textured surfaces that are of high functional significance for tribological purposes. For practical reasons, there is a need to describe such surfaces using only a small number of parameters. Which of them represent surface details the best is still an open issue. To find out which parameters can be the most suitable in that case, three groups of machined textured surfaces were prepared. They were plateau-honed cylinder surfaces made of gray cast iron, steel, and bronze surfaces with isolated dimples and steel surfaces after abrasive blasting followed by lapping. All of them were measured by means of a white light interferometer. Different parameters and relationships were evaluated and based on them correlation and regression analyses were used. The basic description contained statistically independent parameters that can be used in production control, while the wider description in scientific research. In general, parameters of random surfaces were more intercorrelated than those of surfaces with isolated dimples. As was found for the basic description of random two-process surfaces, five parameters were enough while description of textured surfaces with isolated oil pockets needed six. In wider, scientific description, regardless the surface type seven parameters contained the necessary information about the surface. It was also proved that a pair of parameters, the emptiness coefficient Sp/Sz and Sq/Sa, can describe the shape of the ordinate distribution of machined textured surfaces better than, for example, skewness Ssk and kurtosis Sku, commonly used for that purpose.

## 1. Introduction

The surface texture created in the machining process influences the tribological properties of the friction pairs such as contact friction, lubrication, and wear [1,2]. Therefore, the assessment of machined surfaces is important. Typically, machined surfaces are characterized by only one height parameter, typically Ra (arithmetical mean of absolute roughness height). However, this kind of description is not enough for two-process and generally multi-process textures. The plateau-honed cylinder texture is a typical example of two-process surfaces, because it is created by two processes: final honing and plateau honing. The plateau-honed texture resembles the surface created during running-in. This similarity leads to a shorter run-in duration and wear than a one-process honed surface [3]. The plateau-honed cylinder surface combines the good frictional properties of smooth structures and the possibility of maintaining the oil of porous structures. Reference [4] presents a review on the functional importance of plateau-honed cylinder surface texture.

The plateau-honed cylinder surface is an example of textured surfaces, containing multiple engineered features (valleys, individual oil pockets), which are manufactured to improve the functionality [5]. The advantage of textured surfaces over untextured surfaces is well documented. These surfaces minimize friction and decrease wear intensity and tendency for seizure [6,7]. Tribological impacts of surface texturing are visible in unidirectional [8] and bidirectional sliding [9]. Oil pockets are typically spherical, however, other shapes are also possible [10,11]. The beneficial effect of isolated dimples is greater than that of valleys. Among recent scientific contributions, Yin et al. [12] obtained reduction in friction of a piston ring/liner pair, especially at low temperature due to laser texturing of a cylinder liner, up to 38%. Abril et al. [13] found that the increase in dimple density led to the increase in the lubricant film thickness and reduction in the friction force between the piston rings and the cylinder liner in a starved lubrication regime. Xu et al. [14] selected the optimal depth of micro-textures for compliant foil gas seals. The inverted triangular texture presented the best sealing performance. Khaskhoussi et al. [15] studied the effect of surface laser texturing on the wettability of lead-free bronze and lead bronze coatings. The textured surface served as an oil reservoir due to its good oleophilic behavior, leading to superior wear resistance.

As the surface of the cylinder after plateau honing is complicated, several methods were developed to characterize it. The basic methods were related to the material ratio curve, which is a cumulative ordinate distribution. There are five parameters that characterize this curve from the Sk family [16]: the reduced peak height Spk, the reduced valley depth Svk, the core roughness depth Sk, and the material ratios Sr1 and Sr2. The profile versions of these parameters are useful in production control of cylinder liners [17]. They can be used for description of various types of surfaces [18].

The other proposal of material ratio curve description (Sq group) contains three parameters. The plateau root mean square roughness (Spq) parameter is the slope of a regression line executed through the plateau part, and valley root mean square roughness (Svq) through the valley portion. The Smq parameter is the material ratio of plateau-to-valley transition [16]. In this approach, standard deviation is the unit of the material ratio [19,20].

A set of parameters from the V group is the third proposal of material ratio curve description of plateau-honed cylinder surfaces. They are: Vmp—maximum material volume, Vmc—core material volume, Vvc—core void volume, and Vvv—dale void volume.

The parameters from Sk, Sq, and V groups were critically reviewed in [16]. The parameters from the Sk family are easily obtained. The Sk parameter is related to oil consumption [21], therefore, it should be minimized. The Svk parameter is related to the volume of lubricant reservoir in surface topography, called oil capacity; therefore, it should be kept in a defined range. The main problem is that the oil capacity is related to the minimum slope of the material ratio curve, therefore, it can be falsely estimated. Furthermore, the functional importance of the Spk or especially the Sr1 parameter is small. The other deficiencies of the Sk group were presented in [16]. The three parameters from the Sq group are more based on theory. However, this method is difficult to apply. Parameters from the V group are similar to those from Sk family—the surface is divided into three parts: peak, core, and valley. The boundaries of these portions are arbitrarily given, in calculations of parameters from the V group. Combining the Sk and V methods [22] is a good idea.

The parameters of the material ratio curve are used in industry. However, there are other methods that characterize the plateau-honed surfaces of cylinder liners. Various surface features can be characterized, such as the honing angle [23,24] or the distribution of honing valleys in two directions [25,26]. Applications of typical filters, such as Gaussian filter, to plateau-honed cylinder surfaces lead to surface distortion, therefore special filters such as valley suppression filters or robust filters should be used [27,28,29].

Textured surfaces with isolated oil pockets can also be described by parameters characterizing the material ratio curve. They are often described by pit area ratio (density of oil pockets), depths and widths of dimples, and roughness height of texture free of dimples, characterized typically by the Ra parameter. The shape and array of oil pockets contain additional information. The pit area ratio can be estimated on the basis of the material ratio curve [16]. Typically, surfaces with isolated dimples have a random–deterministic character (the valley part is deterministic). However, the plateau part can also be deterministic. They can be two-process or three-process surfaces; in the last case, surfaces without dimples can have a two-process character or the bulges can be removed by grinding, polishing, or lapping (third process).

Textured surfaces cannot be characterized using only one parameter. The selected parameters should be independent, characterize different surface features, be easily calculated, and have a low sensitivity to measurement errors. The independence of the parameters can be evaluated by the linear correlation coefficient. The articles [30,31,32,33,34,35,36] present applications of this coefficient to describe one-process surfaces. Nowicki [30] proposed two parameters for the inspection of roughness profiles. Gorlenko [31] obtained strong correlation between amplitude parameters of roughness profiles. Terry and Brown [32] analyzed statistical dependencies among parameters of roughness profiles after grinding. Ham and Powers [33] selected parameters that describe roughness profiles after single point incremental forming. Etxeberria et al. [34] proposed six areal parameters for characterizing biomaterial surfaces. Qi et al. [35] performed a correlational study of areal surface texture parameters of machined surfaces. Fecske et al. [36] studied correlations among amplitude parameters of modeled surfaces with Gaussian ordinate distribution. Plateau-honed surfaces were analyzed in [37,38], while textured surfaces with isolated dimples created by the burnishing technique were studied in [39]. However, in [37] the correlations among profile parameters were mainly studied. In [38], only surfaces of honed cylinder liners with diamond stones were analyzed. The paper [40] presents a description of two-process surfaces of random and random–deterministic characters. However, not only machined surfaces, but also worn surfaces, such as piston skirt surfaces, were studied in [34].

A textured surface is highly skewed; the Ssk parameter is negative and Sku parameter is higher than 3, the emptiness coefficient Sp/Sz is smaller than 0.5 and the Sq/Sa ratio is higher than 1.25 (Sp—maximum peak height, Sz—maximum height of the surface Sp—maximum peak height, Sz—maximum height of the surface, Sq—rms. height of the surface, Sa—arithmetical mean of the absolute surface heights).

Parametric description of machined surfaces should be accompanied by visual inspection.

One can see from the literature review that general machined textured surfaces containing cavities of various types (valleys and dimples) have not been described yet. Isotropic surfaces after vapor blasting followed by lapping have not been analyzed. This work aims to fill these gaps.

In this work, a proposal of the description of textured areal surfaces based on linear correlation and regression analyses will be presented. Special types of surfaces will be analyzed separately: plateau-honed surfaces, surfaces having isolated dimples, and surfaces after abrasive blasting followed by lapping. In the final part of this paper, a description of general textured machined surfaces containing cavities of various types will be proposed.

This work will be helpful for selection of parameters characterizing machined textured surfaces. The basic description which contains independent parameters can be used in control of machining processes. Wider parametric characterization can be applied in scientific works.

## 2. Materials and Methods

Three groups of measured machined textured surfaces were analyzed. The number of surfaces in each group was twenty. Diversified textures were obtained within each group. The plateau-honed cylinder liners were made of gray cast iron with clearly created phosphorus eutectic. This material is frequently used for diesel engines of trucks. It has a tensile strength of 320 MPa and a hardness of 218 HB. The cylinder liner surfaces were initially rough-bored. Then they were rough-honed using an SZS-250 hone machine made by the WMW firm using a D151/112/x44/35 diamond stick with grain dimension between 125 and 150 µm. The maximum height of surface roughness profiles after rough honing was near 20 µm. The last two operations, finish honing and plateau honing, were carried out by diamond or ceramic stones using the Gehring honing machine, model SZS150.M. D76/112/x44/25 diamond stones were used during finish honing, and D15/118/x44/75 stones during plateau honing. Ceramic stones of 8 × 10 × 150 GS150-60GV4S were applied during the finish honing and 8 × 10 × 100 YC500-R5-V during the plateau honing. The cutting zone was cooled using HON 15 fluid during rough, finish and plateau honing. The plateau honing time was between 8 and 24 s. The texture of the liners was changed as a result of application of various stones (diamond or ceramic) or varying plateau honing time. They were anisotropic, cross-hatched, two-process random textures.

The samples in the second group were made of steel, gray cast iron, and bronze. They contained oil pockets, made by different techniques: abrasive jet machining and burnishing. Some textured surfaces were made from 42CrMo4 steel with 50 HRC hardness after the heat treatment. They were created using abrasive jet machining. In abrasive jet machining, work pressure was 0.6 MPa, nozzle diameter was 8 mm, the distance between the nozzle and machined surface was 100 mm. Aluminum oxide was abrasive with grain dimensions between 75 and 106 µm. Before texturing, disc surfaces were polished, lapped, milled, or ground. Oil pockets created by abrasive jet machining had various shapes: spherical, oval, triangular, chevron, and had various patterns (spiral, radial, and others). After plateau honing, the dimples were created on the surface made from gray cast iron using impulsive burnishing. In this method, a special ending acted as a hammer to form dimples on the cylinder liner surface. Spherical dimples of random patterns were created. Spherical dimples were also created on the block samples, made from bronze CuSn10P of 138 HB hardness by the burnishing technique. Percussive burnishing with electromagnetic drive was used. The machine employed the impulse method, using kinetic energy of working elements. Dimples were created on inner cylinder surfaces of a diameter of 30 mm and length up to 85 mm. Before burnishing, block specimens were precisely turned using a CNC machine.

Textured surfaces were characterized by surface topography parameters and also pit area ratio, depth, and width of dimples. The surfaces free of dimples obtained after polishing, lapping, grinding, honing, and milling had various heights. Textured surfaces were two- or three-process topographies. They were created to improve tribological characteristics of sliding elements.

**Figure 1:**
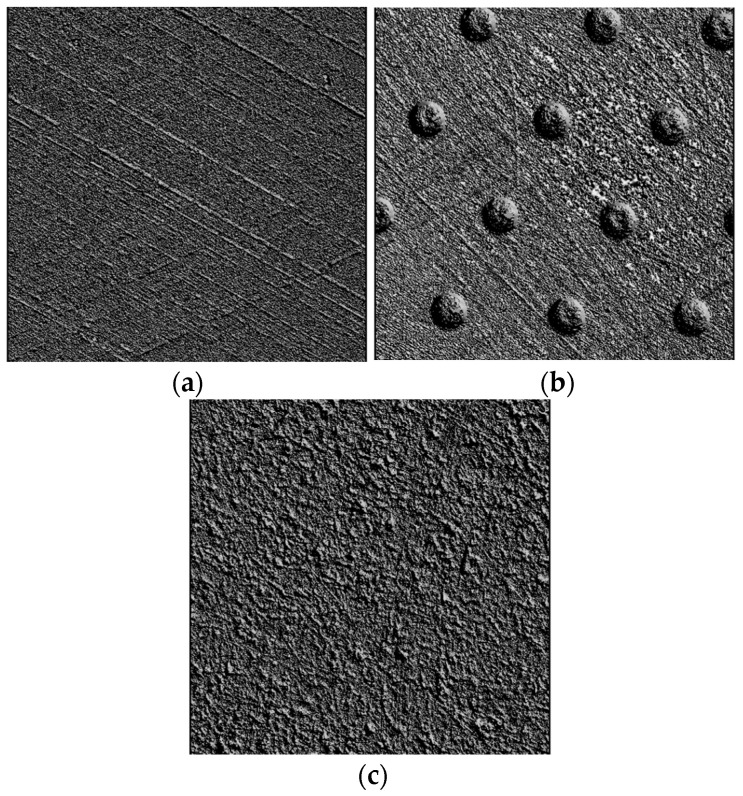
Photo images of selected surfaces after plateau honing (**a**), with isolated dimples (**b**), and after vapor blasting followed by lapping (**c**).

The materials used were typical for plateau-honed cylinder surfaces and surfaces with isolated oil pockets. The third group of samples made from 42CrMo4 steel of 40 HRC hardness consisted of surfaces after abrasive blasting followed by lapping, which is a method of machining leading to good tribological properties [41]. Aloxite with 100 and 120 mm granulations was used during vapor blasting; the air pressure was between 0.2 and 0.6 MPa; and the machining time was between 60 and 120 s. Cabin KIS-900 with nozzle diameter of 5 mm was used for vapor blasting. Lapping was performed using an FS 420 machine with P800–P2000 abrasive papers. They were two-process random isotropic surfaces; the values of the Str parameter (texture aspect ratio) were higher than 0.76. Figure 1 presents photo simulations of selected analyzed surfaces.

The parameters were assumed to be highly correlated when the absolute value of the linear coefficient of correlation between them was higher than 0.7. Surface topographies were measured using the same equipment: Talysurf CCI Lite white light interferometer of 0.01 nm vertical resolution. Conditions of measurement and analysis were the same for all sixty surfaces analyzed. The measurement area of 3.29 × 3.29 mm contained 1024 × 1024 data points. Curvatures were removed by polynomial of the second level; flat surfaces were only leveled. Digital filtration was not used. Non-measured points were filled. Prior to parameter calculations, the spikes were eliminated. Maximum percentage of non-measured points was 10%.

**Figure 2:**
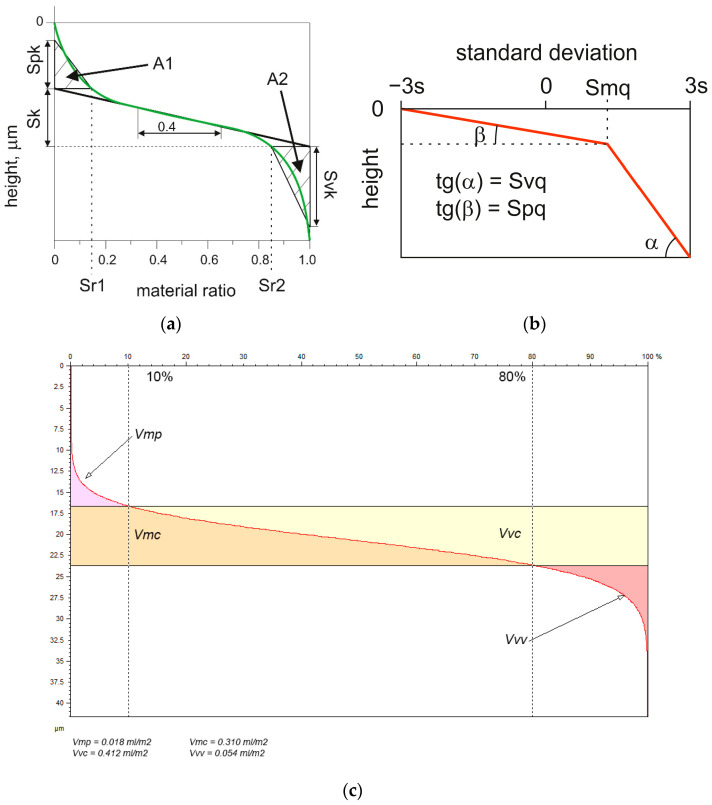
Parameters from the Sk (**a**), Sq (**b**), and V (**c**) groups.

The following parameters from ISO 25178:2 standard were studied: height parameters: Sq, Ssk, Sku, Sp, and Sv—the maximum depth of the valley, spatial parameters: Str, the correlation length Sal and hybrid parameter rms. slope Sdq. The parameters from Sk (except for Sr1 and Spk of small functional significances) and V groups (Vmc and Vvv) were also included, parameters from the Sq family were used only for two-process random surfaces (after plateau honing and after abrasive blasting followed by lapping). Figure 2 presents graphical interpretations of parameters from Sk, Sq, and V groups. Among feature parameters, the density of the peaks Spd and the mean peak curvature Spc were also considered. The pair Pp/Pz and Pq/Pa is a better alternative of the pair Psk and Pku to characterize unfiltered profiles of highly skewed surfaces. Sp, Sz, Sq, Sa, Ssk, and Sku are three-dimensional extensions of profile parameters Pp, Pz, Pq, Pa, Psk, and Pku. Therefore, the emptiness coefficient Sp/Sz and Sq/Sa were also analyzed. The analysis of measured surfaces was carried out using TalyMap 6 software.

## 3. Descriptions of Various Groups of Textured Surfaces

### 3.1. Plateau-Honed Cylinder Liner Surfaces

**Figure 3:**
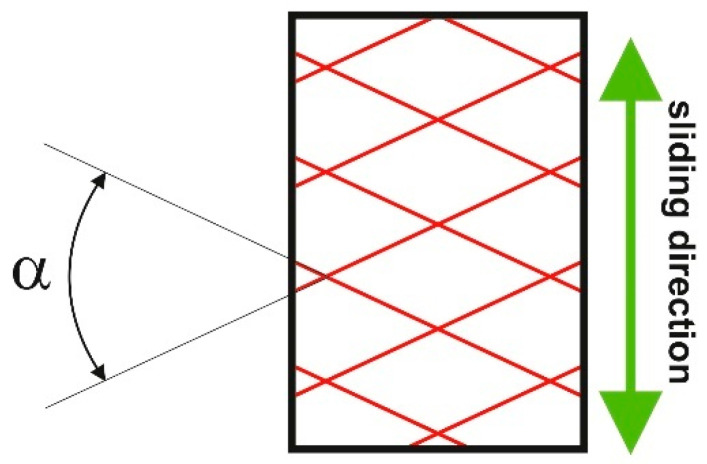
Honing angle α.

The plateau-honed surfaces of the cylinder liners were characterized by low values of the skewness Ssk (between −3.7 and −1.3) and high values of the kurtosis Sku (between 5 and 23). The Sq/Sa ratio was higher than 1.3 and lower than 1.7, while the emptiness coefficient Sp/Sz was relatively small (between 0.17 and 0.29). The amplitude of the roughness, determined by the Sq parameter, ranged from 0.33 to 1.1 µm. The surfaces were anisotropic, the texture aspect ratio Str was smaller than 0.04. The correlation length Sal was in the range 0.014–0.02 mm, the peak density Spd was between 650 and 1550 /mm^2^. The honing angle α (Figure 3) was between 54 and 58 degrees.

**Figure 4:**
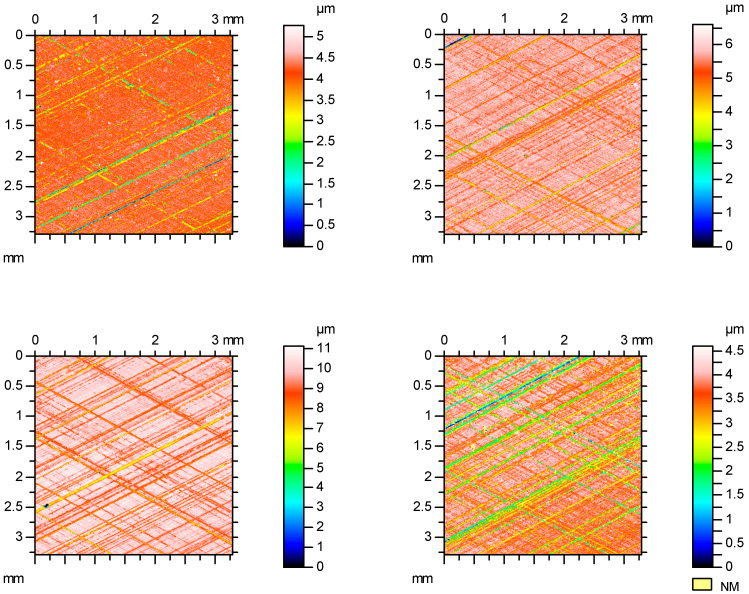
Contour plots of the plateau-honed cylinder surfaces.

**Figure 5:**
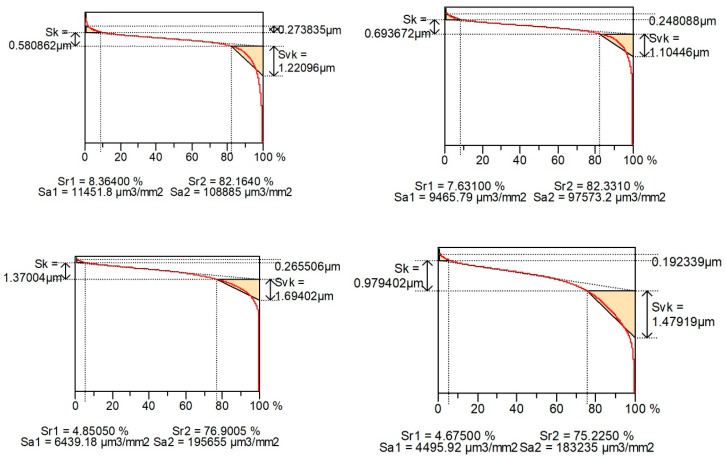
Material ratio curves of surfaces shown in Figure 4.

Figure 4 shows examples of contour plots of the cylinder surfaces after plateau honing, while Figure 5 presents their material ratio curves. Smaller values of Spk than Svk and a larger area under the material ratio curve than above this curve are characteristic features of the two-process surface.

Table 1 lists the coefficients of linear correlation between the selected parameters of the plateau-honed cylinder surfaces.

Height parameters Sq and Sp were strongly intercorrelated, the linear correlation coefficient r between them was 0.92. However, the parameter Sv was not strongly correlated with Sq and Sp. From among the V family, the Vvv parameter was highly correlated with the Vmc parameter (r = 0.71). Within the Sk group, low correlation was found between the Sk and Sk parameters.

The standard deviation of plateau height Spq and the standard deviation of the valley height Svq were also studied, these parameters were statistically independent (r = 0.17). The Smq parameter was independent of the Spq and Svq heights.

**Figure 6:**
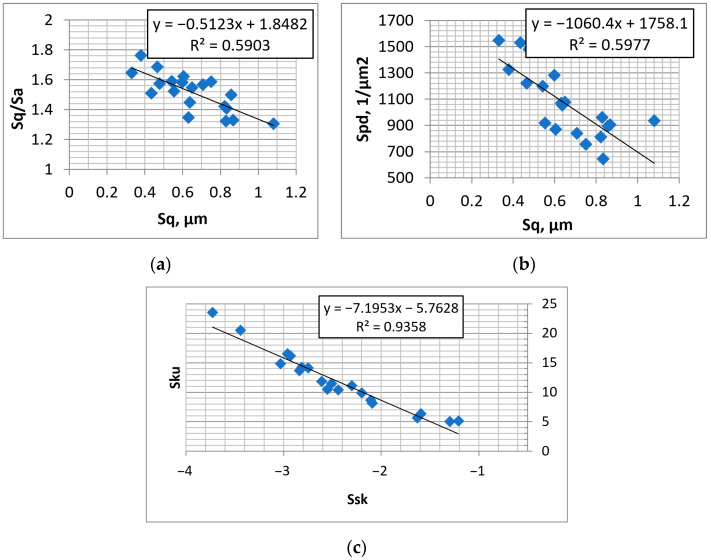
Dependencies between parameters: Sq-Sq/Sa (**a**), Sq-Spd (**b**), and Ssk-Sku (**c**) of plateau-honed cylinder surfaces.

One can see from the analysis of Table 1 that most of the height parameters and slopes were inversely proportional to the peak density Spd. The Ssk and Sku parameters were highly correlated, in contrast to the Sq/Sa and Sp/Sz pair. The inverse proportionality between the Sku or Sq/Sa parameters and surface height is interesting. Perhaps the lower roughness height corresponded to comparatively deeper valleys. The Spq parameter was proportional only to Sp, rms. slope Sdq, Vmc, and Sk parameters, it was also proportional to Spk. A high correlation between the standard deviation of the plateau height and core roughness height was also observed in [34,38]. The material ratio Smq was proportional to Sku and Sq/Sa and inversely proportional to Vmc. The Sp/Sz ratio was highly correlated only with the Ssk parameter. Sal and Str were independent parameters. As the Sv parameter was more independent than the Sp parameter, it was selected for the surface basic description, which also contains Sal, Str, Sq/Sa, and Sp/Sz parameters. When the parameters Sv, Sp/Sz are known, one can easily calculate Sz and Sp (Sp + Sv = Sz). The other proposal is related to the two-process random surface modeling. In this modeling, there are the following input parameters: the correlation lengths in perpendicular directions, Spq, Svq, Smq [4] and the honing angle α. As the honing angle in this research was nearly constant, its high correlation with other parameters was not found. When the parameters Str and Sal are known, one can easily calculate the correlation lengths in perpendicular directions. The parameters Spq, Smq, and Svq are mutually interdependent. Therefore, the second basic description contains parameters Spq, Svq, Smq, Sal, and Str. The parameters of the first and second proposals are mutually interdependent. The parameters Spd and Sdq belong to a wider description. They describe different surface features than parameters from the basic description. Figure 6 shows selected dependencies between parameters of the plateau-honed cylinder surfaces.

### 3.2. Surfaces with Isolated Dimples

Textured surfaces with separate dimples were measured and analyzed. They had a typically random (plateau)–deterministic (valley) character. The depth of the dimples ranged from 2 to 20 µm, width from 120 to 600 µm, pit area ratio from 3 to 30%. Larger widths of dimples typically corresponded to larger dimple densities. The surface height, characterized by the Sq parameter, was between 0.45 and 7.5 µm. Surfaces were negatively skewed—the Ssk parameter was between −9.1 and −0.65, however, the Sku parameter ranged between 3.7 and 112. The correlation length Sal was between 0.025 and 0.24 mm, the Str parameter was between 0.03 and 0.93, the peak density was between 18 and 1011 mm^2^. One can see that the ranges of the parameters presented were wide.

Taking into account the characteristics of the surfaces analyzed, the parameters of the Sq group were not studied.

Table 2 presents the coefficients of linear correlation between the selected parameters of the textured surfaces with isolated dimples.

The amplitude parameters were interrelated, and the smallest coefficient of correlation was obtained between the Sp and Sv parameters. Unlike the plateau-honed surfaces, the Sq parameter was more related to Sv than to the Sp parameter. This difference was probably related to the typically higher values of the skewness of dimpled surfaces compared to the plateau-honed cylinder surfaces.

Within the Sk group, parameters Sk and Svk were not interrelated. Similarly, within a V family, low correlation was found between Vmc and Vvv. The surface height (especially the Sv parameter, r = 0.88) was proportional to dimple depth, while the correlation length Sal was proportional (r = 0.92) and the Sr2 parameter was inversely proportional (r = −0.86) to the pit area ratio. In [39], a strong correlation was also found between the depth of the dimple and the Sv parameter.

The surface height characterized by the Sq parameter was proportional to the parameters of the V and Sk groups, especially the parameters Vvv and Svk. In contrast to surfaces analyzed previously, height parameters were not correlated with rms. slope Sdq, which was related only to the Sk parameter. The lack of correlation between Sdq and Spc is interesting. It resulted from domination of the surface texture by the valley part. The Sdq parameter characterizes the whole texture, while the Spc parameter characterizes the peak part. The mean peak curvature Spc was proportional to the parameter Sp and a very strong correlation between the Svk and Vvv parameters was found. The skewness Ssk was strongly correlated with the kurtosis Sku and the Sq/Sa ratio. Strong statistical relation was found between the Sku parameter and Sq/Sa. The emptiness coefficient Sp/Sz, peak density Spd, and texture parameter Str were statistically independent. In [39], a high correlation was found between Sal and Str parameters, which probably resulted from similarity between burnished textured surfaces.

Generally, most of the parameters analyzed were not strongly interrelated; this behavior was different to that observed for plateau-honed surfaces. It was caused by the diverse character of surfaces, probably as a result of various methods of oil pocket creation.

The following parameters are included in the basic description of textured surfaces with isolated dimples: Sv, Sq/Sa, Sp/Sz, Sal, Str, and Sdq. These parameters are mutually independent. The wider description also contains independent peak density Spd.

**Figure 7:**
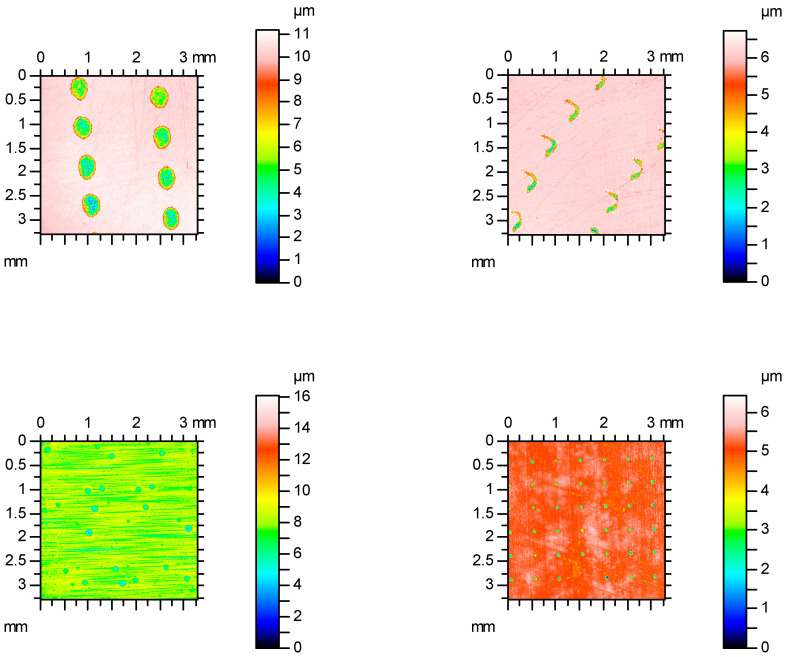
Contour plots of the surfaces containing isolated oil pockets.

**Figure 8:**
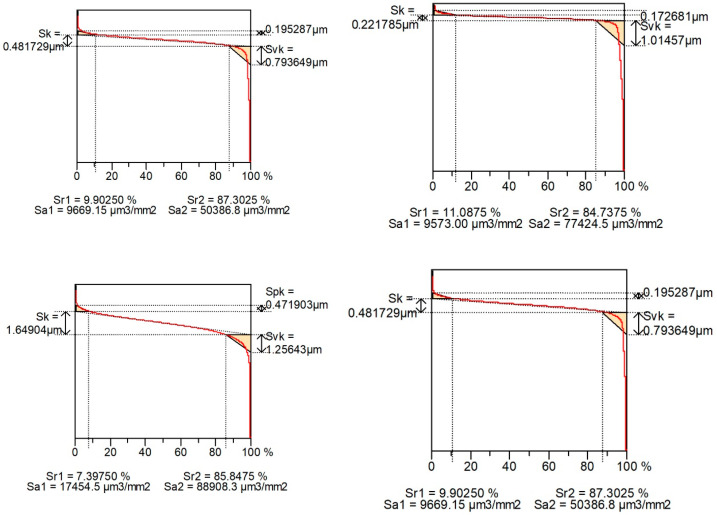
Material ratio curves of surfaces shown in Figure 7.

Figure 7 shows examples of contour plots of surfaces containing isolated oil pockets, while Figure 8 presents material ratio curves of these textures.

**Figure 9:**
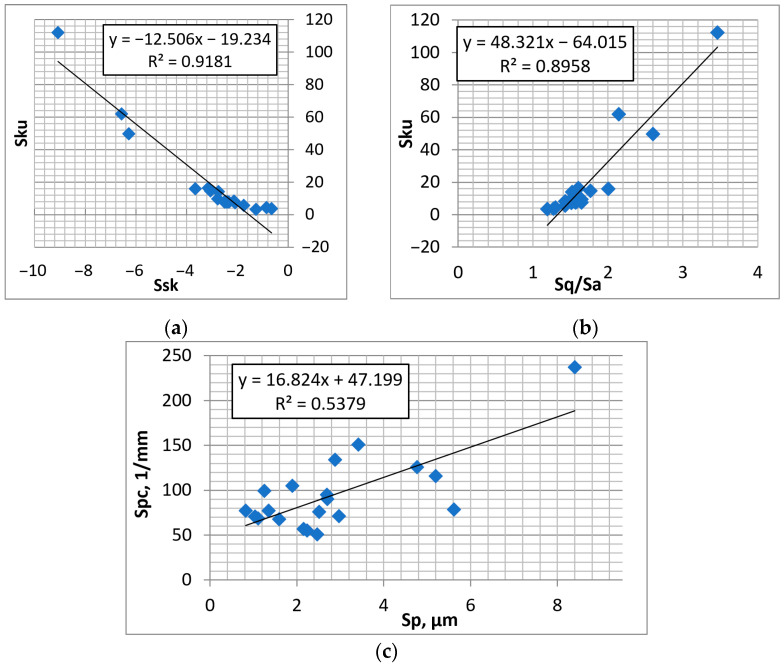
Dependencies between parameters: Ssk–Sku (**a**), Sq/Sa–Sku (**b**), and Sp–Spc (**c**) of textured surfaces containing isolated dimples.

Figure 9 shows selected dependencies between the parameters of textured surfaces that contain isolated dimples.

### 3.3. Surfaces after Vapor Basting Followed by Lapping

Surfaces after vapor blasting followed by lapping were two-process random textures. Surface height, determined by the Sq parameter, was between 0.7 and 2.7 µm. Skewness Ssk ranged from −6.5 to −1,5, while kurtosis Sku was between 6 and 27. The Sq/Sa ratio ranged from 1.33 to 2.2, and the emptiness coefficient Sp/Sz from 0.1 to 0.42. The correlation length was between 0.011 and 0.039 mm, while the peak density was between 34 and 560 mm^2^. Surfaces were isotropic, the Str parameter was in the range 0.76–0.9. Most of the surfaces were more skewed and of bigger roughness height than plateau-honed cylinder surfaces.

Table 3 shows the coefficients of linear correlation between selected parameters of surfaces after vapor blasting followed by lapping.

The amplitude parameters Sq and Sv were interrelated (r = 0.87). Vmc was strongly correlated with Vvv (r = 0.83), and Sk was strongly correlated with Svk (r = 0.83).

The Sq parameter was strongly correlated with the parameters Vmc and Vvv, Sk and Svk, Sdq and Spc, Spq and Svq. The Sv parameter was statistically related to rms. slope Sdq and parameters characterizing valleys Vvv, Svk, and Svq. The Sp parameter was strongly correlated with the mean peak curvature Spc, standard deviation of the plateau height Spq, and the emptiness coefficient Sp/Sz. The last dependence is interesting. It is probably related to the independence of the Sp parameter from other amplitude parameters—proportionality between Sp and Sp/Sz parameters was also obtained for surfaces from the plateau-honed cylinder liner, however, in that case the r coefficient was smaller (0.6). The skewness Ssk was inversely proportional to the kurtosis Sku. Kurtosis was related to the Sq/Sa ratio, but skewness was related to the emptiness coefficient, however, in the last case the r coefficient was not high (0.65). The Sdq parameter was statistically related to the Vmc, Vvv, Sk, Svk, Spq, and Spc parameters. The Spc parameter was proportional to most of the parameters that describe the amplitude. A strong correlation was found between the parameters that characterize the valleys: Sv, Vvv, Svq, and Svk. The Spq parameter was proportional to Sq, Sp, Sdq, Vmc, Vvv, Spc, Sk, and Svk. The Smq material ratio was proportional only to Sq/Sa, which was also strongly correlated with skewness Ssk and kurtosis Sku. Spd, Str, and Sal were independent parameters.

The surfaces after vapor blasting followed by lapping were similar to plateau-honed textures. Therefore, the same parameters can characterize both of them; first set: Sv, Sq/Sa, Sp/Sz, Sal, and Str and second set: Spq, Svq, Smq, Sal, and Str. The parameters within these groups were mutually independent. The wider description also contains Sdq and Spd.

**Figure 10:**
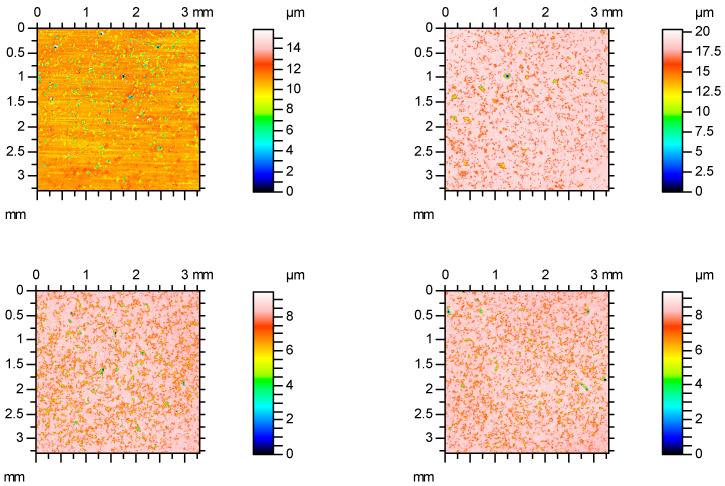
Contour plots of the surfaces after vapor blasting and lapping.

**Figure 11:**
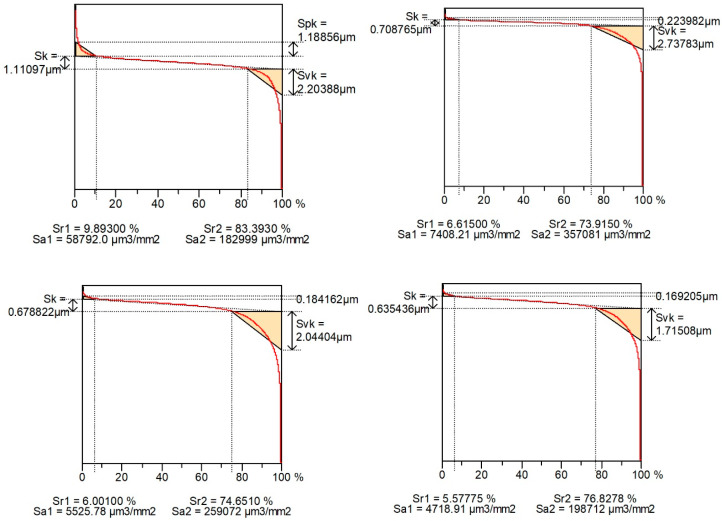
Material ratio curves of surfaces shown in Figure 10.

Figure 10 shows examples of surface contour plots after vapor blasting and lapping, and Figure 11 presents material ratio curves of these surfaces.

**Figure 12:**
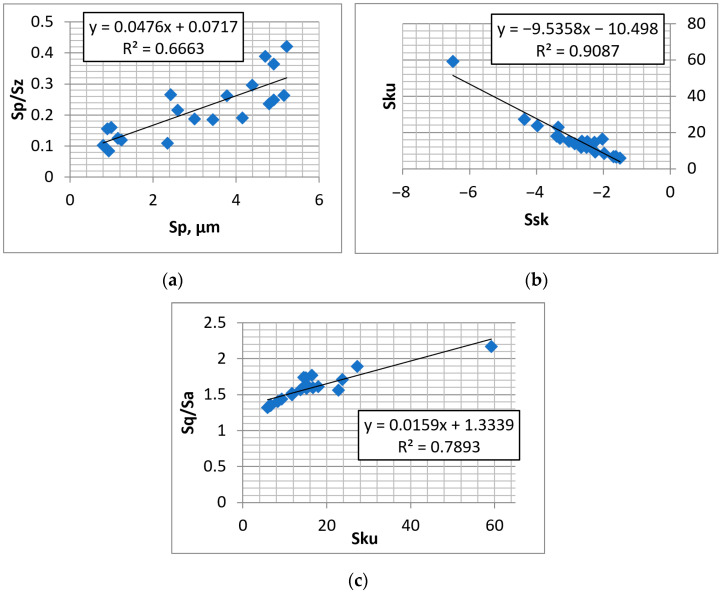
Dependencies between parameters: Sp–Sp/Sz (**a**), Ssk–Sku (**b**), and Sku–Sq/Sa (**c**) of surfaces after abrasive blasting followed by lapping.

Figure 12 presents selected dependencies between the surface parameters after abrasive blasting followed by lapping.

## 4. Description of Textured Machined Surfaces

Seven surfaces after plateau honing, seven surfaces with isolated oil pockets, and six surfaces after abrasive blasting followed by lapping were analyzed. The surface height, characterized by the Sq parameter, was between 0.5 and 7.5 µm. The surfaces were highly skewed, the Ssk parameter was between −9.1 and −1.3. The emptiness coefficient was between 0.08 and 0.36. The Sku kurtosis ranged between 3.2 and 112, while the Sq/Sa ratio between 1.31 and 2.17. The correlation length Sal was between 0.014 and 0.43, while the peak density was between 34 and 1530/mm^2^. The Str parameter ranged between 0.012 and 0.9. These surfaces were characterized by wide ranges of spatial parameters Sal and Str and of peak density Spd.

Table 4 presents the values of the coefficient of linear correlation between selected parameters of the textured surfaces. This table is very similar to that obtained for surfaces with isolated oil pockets. The height parameters were interrelated. This was caused by the high range of the amplitudes of surface textures. The Sq parameter was more correlated with the Sv parameter than with the Sp parameter. Most of the amplitude parameters were strongly correlated with most of parameters from the V and Sk groups. In particular, there were high correlations among parameters that characterized the valley part: Sv, Vvv, and Svk. Height parameters were not strongly correlated with rms. slope Sdq. However, the Spc parameter was proportional to the parameters related to peak and core parts Sp and Sk. The parameters Ssk and Sku, Sku and Sq/Sa, and Ssk and Sq/Sa were statistically dependent. Unlike other analyzed surfaces, the peak density Spd was inversely proportional to the Str parameter; this relation was probably caused by the high peak density of anisotropic plateau-honed cylinder surfaces. Sal, Sdq, and Sp/Sz were independent parameters.

Taking this analysis into account, the following parameters were selected to describe two-process surfaces: Sv, Sq/Sa, Sp/Sz, Str, Sal, and Sdq. The wider description also contains independent parameter Spd. The same parameters were selected to describe surfaces with isolated oil pockets.

As surface texture has a very complex nature, in future research more complex models, such as principal component analysis and multiple linear or non-linear regressions, will be used.

**Figure 13:**
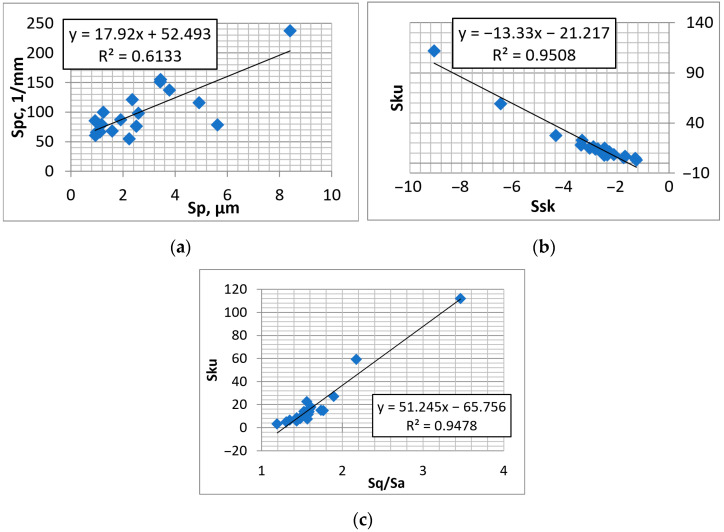
Dependencies between parameters: Sp–Spc (**a**), Ssk–Sku (**b**), and Sq/Sa–Sku (**c**) of the textured surfaces.

Figure 13 presents selected dependencies between the parameters of the textured surfaces.

## 5. Conclusions

1. Random surfaces after plateau honing and after abrasive blasting followed by lapping were characterized by higher mutual correlations of areal texture parameters than surfaces with isolated dimples. Therefore, the basic description of random two-process surfaces contains five parameters, while characterization of textured surfaces with isolated oil pockets contains six parameters.
2. The parameters of two sets constitute the basic description of both types of random two-process surfaces. The first set contains the following parameters: Sv, Sq/Sa, Sp/Sz, Sal, and Str. The second set is related to two-process surface modeling and contains parameters Spq, Svq, Smq, Sal, and Str. Sdq and Spd are included in the wider description.
3. The following parameters are included in the basic description of textured surfaces with isolated dimples: Sv, Sq/Sa, Sp/Sz, Sal, Str, and Sdq. The wider description also contains parameter Spd.
4. For the group of general textured surfaces, the coefficients of linear correlation between parameters were similar to those obtained for surfaces with isolated dimples. Therefore, the description of general textured surfaces contains the same parameters as characterization of surfaces with isolated oil pockets.
5. The pair of parameters Sp/Sz and Sq/Sa better describes the shape of the ordinate distribution of textured surfaces than skewness Ssk and kurtosis Sku. The proposed parameters are mutually independent, in contrast to Ssk and Sku.

## Figures and Tables

**Table 1:** Coefficients of linear correlation between the selected parameters of the plateau-honed cylinder surfaces, the strong correlations are marked by yellow color.

	Sq	Sp	Sv	Ssk	Sku	Sal	Str	Sdq	Vmc	Vvv	Spd	Spc	Sk	Svk	Spq	Svq	Smq	Sq/Sa	Sp/Sz
Sq	1																		
Sp	0.92	1																	
Sv	0.69	0.43	1																
Ssk	0.7	0.81	0.14	1															
Sku	−0.73	−0.77	−0.22	−0.97	1														
Sal	0.52	0.36	0.69	0.23	−0.34	1													
Str	0.42	0.42	0.27	0.47	−0.45	0.45	1												
Sdq	0.99	0.95	0.63	0.73	−0.73	0.38	0.44	1											
Vmc	0.93	0.94	0.48	0.85	−0.8	0.27	0.37	0.94	1										
Vvv	0.91	0.72	0.79	0.5	−0.61	0.66	0.43	0.85	0.71	1									
Spd	−0.77	−0.52	−0.93	−0.31	0.37	−0.68	−0.27	−0.7	−0.62	−0.84	1								
Spc	0.9	0.87	0.69	0.57	−0.6	0.63	0.54	0.91	0.79	0.85	−0.68	1							
Sk	0.87	0.96	0.39	0.84	−0.74	0.26	0.36	0.9	0.94	0.6	−0.5	0.75	1						
Svk	0.88	0.68	0.83	0.44	−0.56	0.67	0.39	0.84	0.68	0.97	−0.85	0.86	0.55	1					
Spq	0.65	0.8	0.35	0.67	−0.56	0.2	0.23	0.7	0.73	0.38	−0.44	0.61	0.83	0.4	1				
Svq	0.14	0.02	0.45	−0.34	0.32	0.14	−0.08	0.15	−0.07	0.2	−0.29	0.2	0.04	0.25	0.17	1			
Smq	−0.68	−0.62	−0.39	−0.68	0.72	−0.48	−0.36	−0.65	−0.75	−0.63	0.52	−0.62	−0.54	−0.61	−0.29	0.45	1		
Sq/Sa	−0.77	−0.81	−0.28	−0.97	0.95	0.32	−0.41	0.17	−0.89	−0.57	−0.46	−0.62	−0.83	−0.55	−0.64	0.3	0.76	1	
Sp/Sz	−0.27	0.6	−0.46	0.7	−0.59	−0.24	0.21	−0.21	0.48	0.01	0.32	0.25	0.59	−0.06	0.28	−0.38	−0.27	−0.56	1

**Table 2:** Coefficients of linear correlation between selected parameters of surfaces that contain isolated oil pockets, the strong correlations are marked by yellow color.

	Sq	Sp	Sv	Ssk	Sku	Sal	Str	Sdq	Vmc	Vvv	Spd	Spc	Sk	Svk	Sq/Sa	Sp/Sz
Sq	1															
Sp	0.78	1														
Sv	0.93	0.73	1													
Ssk	0.3	0.25	0.01	1												
Sku	−0.32	−0.18	−0.01	−0.96	1											
Sal	0.43	0.16	0.29	0.33	−0.41	1										
Str	0.01	−0.09	0.03	−0.3	0.17	0.62	1									
Sdq	0.3	0.62	0.23	0.43	−0.24	−0.23	−0.53	1								
Vmc	0.74	0.77	0.61	0.4	−0.32	0.37	−0.18	0.58	1							
Vvv	0.96	0.66	0.91	0.28	−0.33	0.42	0.06	0.15	0.55	1						
Spd	−0.5	−0.4	−0.53	0.25	−0.12	−0.49	−0.55	0.42	−0.2	−0.52	1					
Spc	0.31	0.73	0.34	−0.06	0.15	−0.1	−0.05	0.66	0.53	0.14	−0.12	1				
Sk	0.69	0.85	0.57	0.36	−0.28	0.04	−0.28	0.76	0.86	0.51	−0.09	0.74	1			
Svk	0.97	0.68	0.91	0.29	−0.33	0.5	0.07	0.15	0.62	0.98	−0.54	0.19	0.54	1		
Sq/Sa	−0.3	−0.18	0.1	−0.96	0.95	−0.4	0.19	−0.35	−0.39	0.35	−0.25	0.16	−0.32	0.26	1	
Sp/Sz	−0.21	0.25	−0.33	0.5	−0.35	−0.31	−0.52	0.53	0.13	0.22	0.35	0.27	−0.29	−0.28	−0.34	1

**Table 3:** Coefficients of linear correlation between selected parameters of surfaces after vapor blasting followed by lapping, the strong correlations are marked by yellow color.

	Sq	Sp	Sv	Ssk	Sku	Sal	Str	Sdq	Vmc	Vvv	Spd	Spc	Sk	Svk	Spk	Svq	Smq	Sq/Sa	Sp/Sz
Sq	1																		
Sp	0.55	1																	
Sv	0.87	0.39	1																
Ssk	0.42	0.64	0.04	1															
Sku	−0.44	−0.47	−0.08	−0.95	1														
Sal	0.57	−0.09	0.65	−0.3	0.2	1													
Str	0.28	0.03	0.15	−0.11	0.08	0.5	1												
Sdq	0.93	0.65	0.7	0.62	−0.59	0.35	0.25	1											
Vmc	0.92	0.57	0.68	0.59	−0.57	0.43	0.25	0.96	1										
Vvv	0.96	0.46	0.89	0.35	−0.41	0.6	0.25	0.85	0.83	1									
Spd	−0.35	−0.09	−0.64	0.39	−0.39	−0.31	0.02	−0.21	−0.18	−0.36	1								
Spc	0.79	0.77	0.68	0.44	−0.37	0.39	0.17	0.84	0.82	0.7	−0.27	1							
Sk	0.91	0.66	0.66	0.6	−0.54	0.37	0.26	0.96	0.99	0.8	−0.16	0.85	1						
Svk	0.97	0.49	0.89	0.36	−0.4	0.61	0.26	0.86	0.85	0.99	−0.36	0.72	0.83	1					
Spq	0.87	0.72	0.68	0.54	−0.48	0.42	0.2	0.85	0.89	0.78	−0.17	0.87	0.92	0.82	1				
Svq	0.7	0.22	0.87	−0.25	0.21	0.69	0.35	0.45	0.42	0.75	−0.24	0.45	0.42	0.76	0.46	1			
Smq	−0.27	−0.1	0.05	−0.59	0.62	0.1	0.01	−0.5	−0.51	−0.18	−0.3	−0.22	−0.46	−0.16	−0.21	0.36	1		
Sq/Sa	−0.53	−0.34	−0.21	−0.84	0.89	0.02	0.06	−0.69	−0.68	−0.46	0.27	−0.45	−0.69	−0.46	−0.56	0.17	0.77	1	
Sp/Sz	0.03	0.82	−0.15	0.65	−0.42	−0.55	−0.14	0.2	0.11	−0.03	0.2	0.32	0.22	−0.02	0.27	−0.24	−0.05	−0.15	1

**Table 4:** Coefficients of linear correlation between selected parameters of the textured surfaces, the strong correlations are marked by yellow color.

	Sq	Sp	Sv	Ssk	Sku	Sal	Str	Sdq	Vmc	Vvv	Spd	Spc	Sk	Svk	Sq/Sa	Sp/Sz
Sq	1															
Sp	0.8	1														
Sv	0.9	0.76	1													
Ssk	0.21	0.08	−0.14	1												
Sku	−0.21	−0.02	0.12	−0.98	1											
Sal	0.51	0.27	0.31	0.24	−0.21	1										
Str	0.18	0.38	0.35	−0.31	0.29	0.38	1									
Sdq	0.22	0.43	0.31	0.18	−0.16	−0.29	0.25	1								
Vmc	0.83	0.75	0.63	0.38	−0.31	0.56	0.16	0.32	1							
Vvv	0.97	0.7	0.89	0.19	−0.22	0.45	0.12	0.12	0.68	1						
Spd	−0.53	−0.58	−0.67	0.28	−0.25	−0.39	−0.81	−0.22	−0.41	−0.47	1					
Spc	0.44	0.78	0.51	−0.18	0.23	0.01	0.45	0.61	0.53	0.27	−0.46	1				
Sk	0.77	0.83	0.6	0.32	−0.27	0.24	0.11	0.49	0.87	0.64	−0.32	0.74	1			
Svk	0.98	0.71	0.88	0.24	−0.24	0.62	0.17	0.1	0.77	0.98	−0.5	0.28	0.65	1		
Sq/Sa	−0.19	0.01	0.13	−0.95	0.97	−0.2	0.29	−0.18	−0.35	−0.18	−0.23	0.24	−0.27	−0.22	1	
Sp/Sz	−0.14	0.26	−0.33	0.48	−0.32	−0.03	−0.04	0.12	0.09	−0.21	0.19	0.14	0.16	−0.22	−0.24	1

## Data Availability

Not applicable.

## Nomenclature

r	coefficient of linear correlation
Sal	auto-correlation length
Sdq	root mean square slope
Sk	core roughness depth
Sku (Pku)	kurtosis
Smq	material ratio at plateau-to-valley transition
Sp (Pp)	maximum peak height
Spc	mean peak curvature
Spd	peak density
Spk	reduced peak height
Spq	plateau root mean square roughness
Sq (Pq)	root mean square height
Ssk (Psk)	skewness
Str	texture aspect ratio
Sv (Pv)	maximum valley depth
Svk	reduced valley depth
Svq	valley root mean square roughness
Sz (Pz)	maximum height
Vmc	core material volume
Vmp	peak material volume
Vvc	core void volume
Vvv	dale void volume
α	honing angle
